# Cardiopulmonary function in paediatric post-COVID-19: a controlled clinical trial

**DOI:** 10.1007/s00431-024-05421-w

**Published:** 2024-01-09

**Authors:** Isabelle Schoeffl, Roman Raming, Jan-Philipp Tratzky, Adrian P. Regensburger, Calvin Kraus, Wolfgang Waellisch, Regina Trollmann, Joachim Woelfle, Sven Dittrich, Rafael Heiss, Ferdinand Knieling, Annika Weigelt

**Affiliations:** 1 Department of Pediatric Cardiology, University Hospital Erlangen, Friedrich-Alexander-Universität Erlangen-Nürnberg, Loschgestrasse 15, 91054 Erlangen, Germany; 2 School of Clinical and Applied Sciences, Leeds Beckett University, Leeds, LS13HE UK; 3 Department of Pediatrics, University Hospital Erlangen, Friedrich-Alexander-Universität Erlangen-Nürnberg, Loschgestrasse 15, 91054 Erlangen, Germany; 4 Department of Radiology, University Hospital Erlangen, Friedrich-Alexander-Universität Erlangen-Nürnberg, Loschgestrasse 15, 91054 Erlangen, Germany

**Keywords:** $\dot{{\text{V}}}{{\text{O}}}_{2}{\text{peak}}$, Cardiopulmonary exercise testing, Post-COVID, Fitness, Exercise capacity, Physical activity

## Abstract

Recently, the importance of post-COVID-19 in children has been recognized in surveys and retrospective chart analysis. However, objective data in the form of cardiopulmonary exercise test as performed in adults suffering from this condition are still lacking. This study aimed to investigate the cardiopulmonary effects of post-COVID-19 on children and adolescents. In this cross-sectional study (the FASCINATE study), children fulfilling the criteria of post-COVID-19 and an age- and sex-matched control group underwent cardiopulmonary exercise testing on a treadmill and completed a questionnaire with regard to physical activity before, during and after the infection with SARS-CoV-2. We were able to recruit 20 children suffering from post-COVID-19 (mean age 12.8 ± 2.4 years, 60% females) and 28 control children (mean age 11.7 ± 3.5 years, 50% females). All participants completed a maximal treadmill test with a significantly lower $\dot{{\text{V}}}{{\text{O}}}_{2}{\text{peak}}$ in the post-COVID-19 group (37.4 ± 8.8 ml/kg/min vs. 43.0 ± 6.7 ml/kg/min. *p* = 0.019). This significance did not persist when comparing the achieved percentage of predicted $\dot{{\text{V}}}{{\text{O}}}_{2}{\text{peak}}$. There were no significant differences for oxygen pulse, heart rate, minute ventilation or breathing frequency.

*   Conclusion*: This is the first study to investigate post-COVID-19 in children using the cardiopulmonary exercise test. Although there was a significantly reduced $\dot{{\text{V}}}{{\text{O}}}_{2}{\text{peak}}$ in the post-COVID-19 group, this was not true for the percent of predicted values. No pathological findings with respect to cardiac or pulmonary functions could be discerned. Deconditioning was the most plausible cause for the experienced symptoms.

*    Trial registration*: clinicaltrials.gov, NCT054445531, Low-field Magnetic Resonance Imaging in Pediatric Post Covid-19—Full Text View—ClinicalTrials.gov.

**Table:** 

**What is Known:** *• The persistence of symptoms after an infection with SARS-CoV 2, so-called post-COVID-19 exists also in children.* *• So far little research has been conducted to analyze this entity in the pediatric population.*	
**What is New:** *• This is the first study proving a significantly lower cardiopulmonary function in pediatric patients suffering from post-COVID-19 symptoms.* *• The cardiac and pulmonary function appear similar between children suffering from post-COVID-19 and those who don’t, but the peripheral muscles seem affected.*

**What is Known:**

*• The persistence of symptoms after an infection with SARS-CoV 2, so-called post-COVID-19 exists also in children.*

*• So far little research has been conducted to analyze this entity in the pediatric population.*

**What is New:**

*• This is the first study proving a significantly lower cardiopulmonary function in pediatric patients suffering from post-COVID-19 symptoms.*

*• The cardiac and pulmonary function appear similar between children suffering from post-COVID-19 and those who don’t, but the peripheral muscles seem affected.*

## Introduction

Besides having caused millions of cases and thousands of deaths worldwide during acute infection [1], there is growing concern about the long-term effects of SARS-CoV 2 infection [2, 3]. Even though children are less affected by severe COVID-19 than adults [4, 5], more and more data is emerging about the persistence of symptoms, so-called post-COVID-19, also in children [6, 7]. This condition is still insufficiently defined but includes signs and symptoms that persist, develop or fluctuate after SARS-CoV-2 infection for at least 2 months and cannot be explained by an alternative diagnosis [8].

Most of the research being conducted about the symptoms of post-COVID-19 focuses on the adult population with limited information about pediatric patients [5, 6]. The most common symptoms being reported in children are fatigue [6, 9–11], exertional dyspnea [11–13] and exercise intolerance [10, 11, 13, 14]. So far, all studies investigating post-COVID-19 in children have relied on surveys [9–11, 15], chart reviews [13], pulmonary function tests [13, 14] and rarely an occasional 6 min-walk-test (6MWT) [13].

In the adult population many studies have investigated the effects of post-COVID-19 on patients using cardiopulmonary exercise testing (CPET) [16]. CPET remains the standard for measuring exercise capacity on top of aiding in the differential diagnosis of the exercise limitations [17, 18]. Several studies established a reduction in peak oxygen uptake ($\dot{{\text{V}}}{{\text{O}}}_{2}{\text{peak}}$), as well as in the predicted $\dot{{\text{V}}}{{\text{O}}}_{2}{\text{peak}}$ and the $\dot{{\text{V}}}{{\text{O}}}_{2}$ at the first ventilatory threshold [16, 19, 20] in post-COVID-19 patients. However, the causes for this reduction are unclear.

While pulmonary dysfunction has been observed on advanced magnetic resonance imaging [21, 22], one possible explanation is the effect of deconditioning combined with alteration in muscular oxygen utilization [16, 23]. Apart from dysfunctional breathing, chronotropic incompetence and abnormal heart recovery have been observed [16, 24]. Interestingly, cardiac output, indirectly measured as the oxygen pulse (O_2_pulse), is not affected in patients suffering from post-COVID-19 [16].

To this date, no study has investigated the effects of post-COVID-19 in children using CPET. As a consequence of being aware of the long-term sequelae of an infection with SARS-CoV-2, concerns have arisen regarding the return to sports after the disease. Although it is clear that a lack of sports and physical exercise has led to severe consequences for children during the pandemic [25, 26], some authors suggest a prolonged rest period with gradual return to sports after extensive medical testing including cardiac screening [27, 28].

## Material and methods

The study was approved by the Ethics Committee of the University of Erlangen-Nuremberg, FRG (206_21B). All study participants as well as their legal guardians gave written informed consent according to the standards set by the Declaration of Helsinki. The study is part of a bigger study and was registered under clinicaltrials.gov as part of the FASCINATE study (NCT054445531) https://clinicaltrials.gov/ct2/show/NCT05445531?term=FASCINATE&draw=2&rank=2.

### Participants

Participants consisted of children between the age of 5 and 17 years with symptoms of post-COVID-19 as defined by the “Arbeitsgemeinschaft der Wissenschaftlichen Medizinischen Fachgesellschaften” AWMF S1 (step 1) guideline [29]. The comparison group consisted of children and teenagers with proof of SARS-CoV-2 infection but post-COVID-19 criteria [29] not fulfilled. All participants were recruited via media (newspaper, homepage).

The inclusion criteria for the patients in the post-COVID-19 group were:

- Positive SARS-CoV-2 infection confirmed by polymerase chain reaction (PCR)
- Post-COVID-19 criteria according to the German guideline AWMF S1 [29] fulfilled

The inclusion criteria for the comparison group were:

- Proof of SARS-CoV-2 infection
- Post-COVID-19 criteria not met

The exclusion criteria for both groups were:

- Clinical presentation of acute infection either by SARS-CoV-2 or other
- Necessary quarantine
- Pregnancy, lactation
- Known pleural or pericardial effusion
- Critical condition (need for respiratory support, ventilation, oxygen administration, shock, symptomatic heart failure)
- Marked thoracic deformities
- Previous lung surgery
- Injuries that do not allow for physical stress testing
- Suspicion of pulmonary disease
- Inhaled therapy (e.g., steroids or beta-mimetics)
- Immunosuppression
- Any condition that may lead to a respiratory limitation (e.g., pain)
- Obesity (> 97% of age percentile)

Height and weight were measured using a stadiometer and electronic scale (Seca 704 S, Hamburg, Germany).

Extracurricular physical activity was assessed differentiating between the type and amount (in hours per week) of sports performed out of school in the year prior to infection with SARS-CoV-2, as well as at the moment of testing, and the length of a rest period from physical activity due to the infection.

### Measurement of gas exchange

A small, low-dead-space respiratory valve (88 ml) with a size-matched mouthpiece and headgear was used (Metalyzer 3B, Cortex, Leipzig, Germany). During each test, the gas exchange was measured continuously using a breath-by-breath method and averaged over 15 s intervals. We used the following physiological criteria for completion of a valid $\dot{{\text{V}}}{{\text{O}}}_{2}{\text{peak}}$, two of which needed to be met for validation: (1) peak heart rate (peak HR) within 5% of the age-predicted maximum, (2) respiratory exchange ratio (RER) ≥ 1.0 and (3) volitional fatigue [30, 31]. We chose a threshold of 1.00 RER for the completion of a valid $\dot{{\text{V}}}{{\text{O}}}_{2}{\text{peak}}$ as it is difficult to achieve higher RER values when performing CPET on a treadmill with children [32]. The peak oxygen uptake was put in relation to normal values from Kalden et al. [33] for children below the age of 8 and from Bongers et al. [34] for children between 9 and 16 years of age. For adolescents above the age of 16 years, normal values for adults [35] were used.

The V-slope method proposed by Beaver et al. [36] was used by the same experienced researcher to determine the ventilatory thresholds VT_1_ and VT_2_. Plotting $\mathrm{oxygen uptake} (\dot{{\text{V}}}{{\text{O}}}_{2}$) (ml/min) against the logarithm of minute ventilation ($\dot{{V}_{{\text{E}}}}$) (ml/min) and calculating the slope of this linear relation through single regression analysis [30] determined the oxygen uptake efficiency slope (OUES).

The ventilatory response during exercise was assessed using a linear regression function by plotting minute ventilation ($\dot{{V}_{{\text{E}}}}$) against carbon dioxide procution (${\dot{V}}_{{\text{CO}}2}$) without the data above the ventilatory compensation point, and the slope (${\dot{V}}_{{\text{E}}}/{\dot{V}}_{{\text{CO}}2}$) was obtained from the regression line [37].

The breathing reserve represents the percentage of the achieved maximal voluntary ventilation (MVV), which was calculated from the FEV_1_ × 35.

A half-time recovery of $\dot{{\text{V}}}{{\text{O}}}_{2}$ (${T}_{1/2}\dot{{\text{V}}}{{\text{O}}}_{2}$) was assessed during off-transient after peak-graded CPET and was defined as the time needed for $\dot{{\text{V}}}{{\text{O}}}_{2}{\text{peak}}$ to decrease by half [38].

Heart rate recovery was monitored during the first minute of the recovery phase (HRR).

### Cardiopulmonary exercise test

All subjects were equipped with a 12-lead ECG (Custo®, Ottobrunn, Germany) for monitoring heart rate and ECG changes.

An incremental step test on a treadmill (COSMED T 170, COSMED, Italy) was performed for cardiopulmonary exercise testing. We used an age-appropriate treadmill testing protocol derived from a previous study [30]. In this protocol the starting speed is set at 3 km/h, with the following steps set at 6 km/h, 8 km/h, and then an increase of 1 km/h every 2 min. We used an increment of 1% for simulation of a natural environment. All participants were encouraged verbally to run until exhaustion and all tests were performed by the same researchers.

### Statistical analysis

Statistical analysis was performed using Microsoft Excel 2000® for data collection and SPSS 12.0® (SPSS Inc., Chicago, IL) for statistical evaluation. All measured values are reported as means and standard deviations. The Kolmogorow-Smirnov test was used to check for normal distribution. Homogeneity of variance was investigated using Levene’s *F*-test. For normally distributed variables, differences between the former preterm children and their healthy control group were assessed with unpaired *t*-tests; otherwise, the Wilcoxon or the Whitney-Mann *U*-tests were used. Statistical significance was set at *p* < 0.05.

Since the study was part of a bigger study that had been powered to find differences in the magnetic resonance imaging performed in the two groups, we performed Cohen’s *D* as well as the correlation coefficient *r* for the primary outcome, namely the peak oxygen uptake $\dot{{\text{V}}}{{\text{O}}}_{2}{\text{peak}}$, in order to measure the effect size.

## Results

### Subjects

Overall, 20 participants suffering from post-COVID-19, and 28 participants for the comparison group were recruited. There were no significant differences between the patients suffering from post-COVID-19 and the controls with respect to age, height, weight or sex (Table 1).

**Table 1:** Characteristics of the participants as median and interquartile range in brackets

	**Post-COVID-19**	**Control**	***p*-value**
*n*	20	28	
Age (y)	13.5 (11.4–15.6)	12.7 (9.2–15.0)	0.242
Male (%)	40.0%	50%	0.493
Height (cm)	161.8 (151.5–168.5)	158.5 (137–173)	0.538
Weight (kg)	50.1 (39.0–63.0)	48.3 (32.0–60.1)	0.422
Body mass index (kg/m^2)^	19.5 (16.8–23.4)	18.4 (15.5–20.6)	0.203
Body surface area (m^2^)	1.5 (1.3–1.7)	1.5 (1.1–1.7)	0.404

The type and amount of physical activity were also comparable between the two groups except for the rest period from physical activity after SARS-CoV-2 infection which amounted to over 4 weeks in the group suffering from post-COVID-19 compared to 0.3 weeks in the control group (Table 2). Overall, the children had performed significantly less physical activity during the pandemic (2.1 ± 2.7 h) than before (3.5 ± 2.8 h).

**Table 2:** Physical activity profile and data from the spirometry of all participants as median and interquartile range in brackets

	**Post-COVID-19**	**Control**	***p*-value**
Physical activity before pandemic (hours/week)	3.5 (1.1–5.0)	3.5 (1.0–4.9)	0.602
Physical activity during pandemic (hours/week)	1 (0.0–3.0)	1.5 (0.0–3.8)	0.552
Physical activity before infection with SARS-CoV-2 (hours/week)	1 (0.0–4.0)	1.8 (0.0–4.5)	0.628
Duration of rest from physical activity (weeks)	2 (0–5.5)	0 (0–0)	0.015
School transportation by bus/car (%)	40%	40%	0.998
Worse subjective exercise tolerance after infection with SARS-CoV-2 (%)	65%	7%	< 0.001

The children suffering from post-COVID-19 stated a lower exercise tolerance.

### Cardiopulmonary exercise test

The data from the cardiopulmonary exercise test is summarized in Table 3.

**Table 3:** Results from the cardiopulmonary exercise test as median and interquartile range in brackets. BF, breathing frequency; BR, breathing reserve; HRR, heart rate recovery; OUES, oxygen uptake efficiency slope

	**Post-COVID-19**	**Control**	***p*-value**
Peak RER	1.05 (1.01–1.07)	1.06 (1.04–1.08)	0.102
$\dot{{\text{V}}}{{\text{O}}}_{2}{\text{peak}}$(ml/kg/min)	39 (32.0–43.0)	41 (38.3–47.8)	0.019
$\dot{{\text{V}}}{{\text{O}}}_{2}{\text{peak}}$(% of normal value)	93 (72.0–97.8)	94 (80.0–112.0)	0.112
$\dot{{\text{V}}}{{\text{O}}}_{2}{\text{VT}}1$(% of $\dot{{\text{V}}}{{\text{O}}}_{2}{\text{peak}}$)	57 (48.8–59.5)	54.5 (49.5–58.0)	0.235
Peak velocity (km/h)	10 (8.0–12.0)	10 (9.0–12.8)	0.171
$\dot{V}{\text{Epeak}}$(l/min)	64.8 (54.5–86.1)	69.8 (52.7–101.0)	0.411
Peak BF (breaths/min)	56 (49.0–69.0)	64 (55.3–73.8)	0.244
BR (%)	27 (19.3–39.0)	27.5 (7.0–39.8)	0.174
Peak O_2_pulse (ml)	9 (8.0–13.0)	10.5 (8.0–13.0)	0.510
Peak HR (beats/min)	192 (185.0–198.0)	192 (185.3–197.8)	0.434
HRR (beats/min)	− 18.6 (− 21.8 to − 14.6)	− 19 (− 22.5 to − 17.4)	0.263
Chronotropic index	84.5 (79.1–87.9)	82.1 (78.3–90.3)	0.244
$\dot{{\text{V}}}{{\text{O}}}_{2}/P$-slope	11.3 (10.1–13.4)	13.1 (11.9–15.0)	0.057
OUES	2 (1.6–2.5)	2.4 (1.6–2.6)	0.514
${\dot{V}}_{{\text{E}}}/{\dot{V}}_{{\text{CO}}2}$-slope	34.3 (31.8–36.6)	35.6 (11.3)	0.426
${T}_{1/2}\dot{{\text{V}}}{{\text{O}}}_{2}$(s)	90 (70–120)	70 (60–110)	0.154

There was a significant difference with regard to exercise time with the post-COVID-19 group achieving less than the control group (855.6 ± 152.9 s. vs. 969.9 ± 152.9 s.). The achieved peak exertion was comparable between the two groups with similar RER, heart rate reserve (HRR) and breathing reserve (BR). No arrhythmias were discerned either at rest, or during exercise or recovery.

**Fig. 1:**
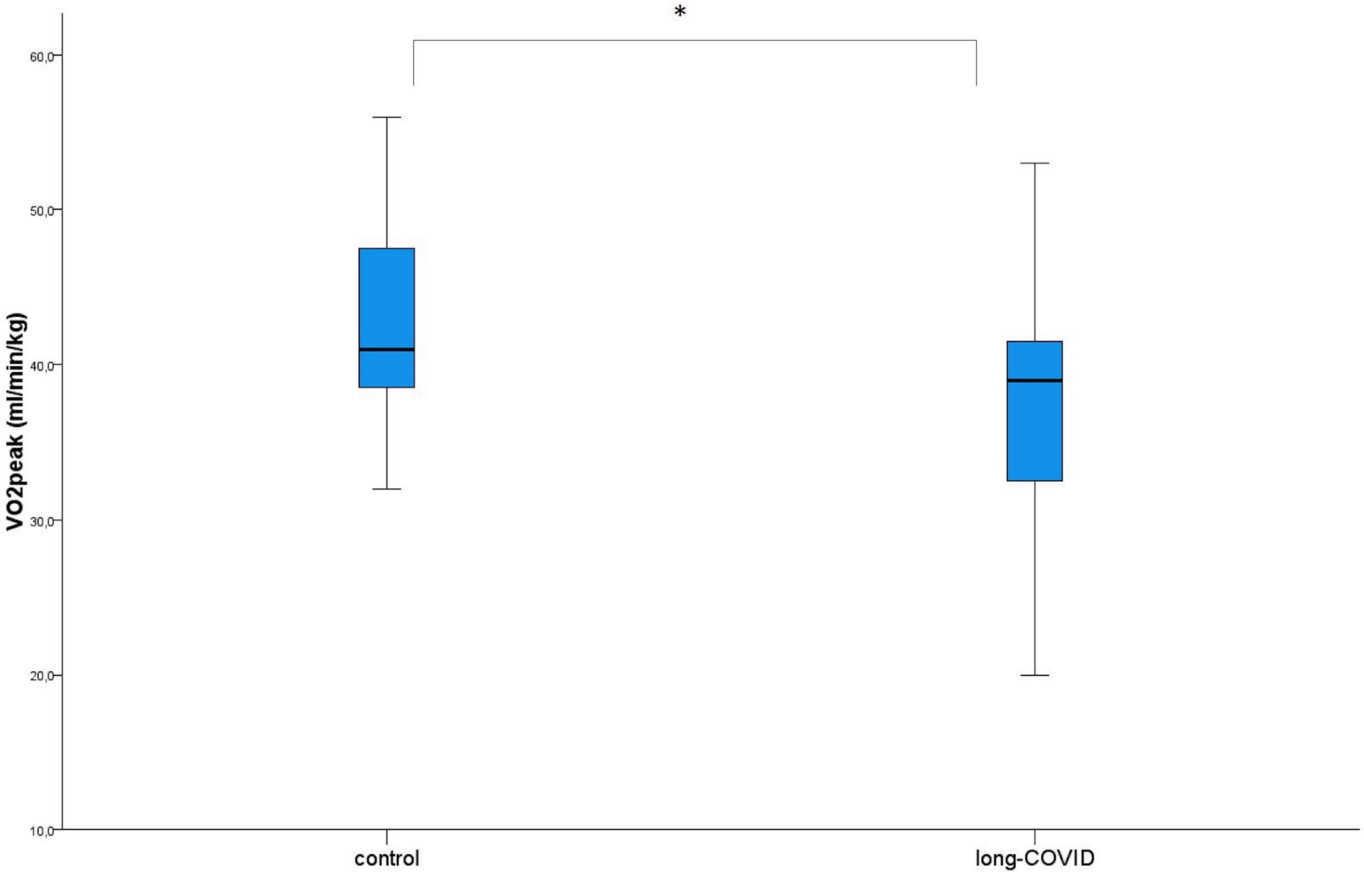
Median, as well as interquartile range, minimum and maximum of the $\dot{{\text{V}}}{{\text{O}}}_{2}{\text{peak}}$ between the children suffering from post-COVID-19 and the control group. The difference was significant

The peak oxygen uptake was significantly lower in the group suffering from post-COVID-19 symptoms (Fig. 1). In a post-hoc analysis, it became apparent that this finding was limited to the female participants (mean values of 33 ± 7.4 ml/kg/min for the girls affected by post-COVID-19 vs. 40.1 ± 5.3 ml/kg/min for the non-affected girls with a *p*-value of 0.005), whereas the boys showed comparable values for $\dot{{\text{V}}}{{\text{O}}}_{2}{\text{peak}}$ (mean values of 43.4 ± 7.2 ml/kg/min for the boys affected by post-COVID-19 vs. 45.7 ± 6.7 ml/kg/min for the non-affected boys, with a *p*-value of 0.451). Furthermore, the predicted $\dot{{\text{V}}}{{\text{O}}}_{2}{\text{peak}}$ did not differ significantly between the two groups, not even when only investigating the girls.

The oxygen uptake at VT1, as well as the percentage of achieved $\dot{{\text{V}}}{{\text{O}}}_{2}{\text{peak}}$ at VT1, was comparable between the two groups and was not affected by sex.

### Pulmonary function

When analyzing the pulmonary measurements from the CPET, there were no significant differences with respect to minute ventilation either at peak exercise $\dot{{\text{V}}}{\text{Epeak}}$ or at the first ventilatory threshold ($\dot{{\text{V}}}{\text{EVT}}1$). Nor were there differences with regard to the breathing reserve (BR).

The slope of ${\dot{V}}_{{\text{E}}}/{\dot{V}}_{{{\text{CO}}}_{2}}$ below the first ventilatory threshold VT1 showed no significant differences between the two groups.

### Cardiac function

All parameters related to cardiac function (Peak O_2_pulse, Peak HR, HRR, ${\dot{V}}_{{\text{E}}}/{\dot{V}}_{{{\text{CO}}}_{2}}$-slope, chronotropic index) were comparable between the two groups (Table 3).

### Peripheral function

Peripheral function represents the third physiologic compartment in the gas transport mechanisms for coupling cellular to pulmonary respiration according to Wasserman [39]. The third compartment represents the mitochondrial function of the muscle [39]. One further parameter for analyzing peripheral function is the ${T}_{1/2}\dot{{\text{V}}}{{\text{O}}}_{2}$, the time needed for $\dot{{\text{V}}}{{\text{O}}}_{2}{\text{peak}}$ to decrease by half (Table 3). There were no significant differences between the two groups in this study.

### Correlations

**Fig. 2:**
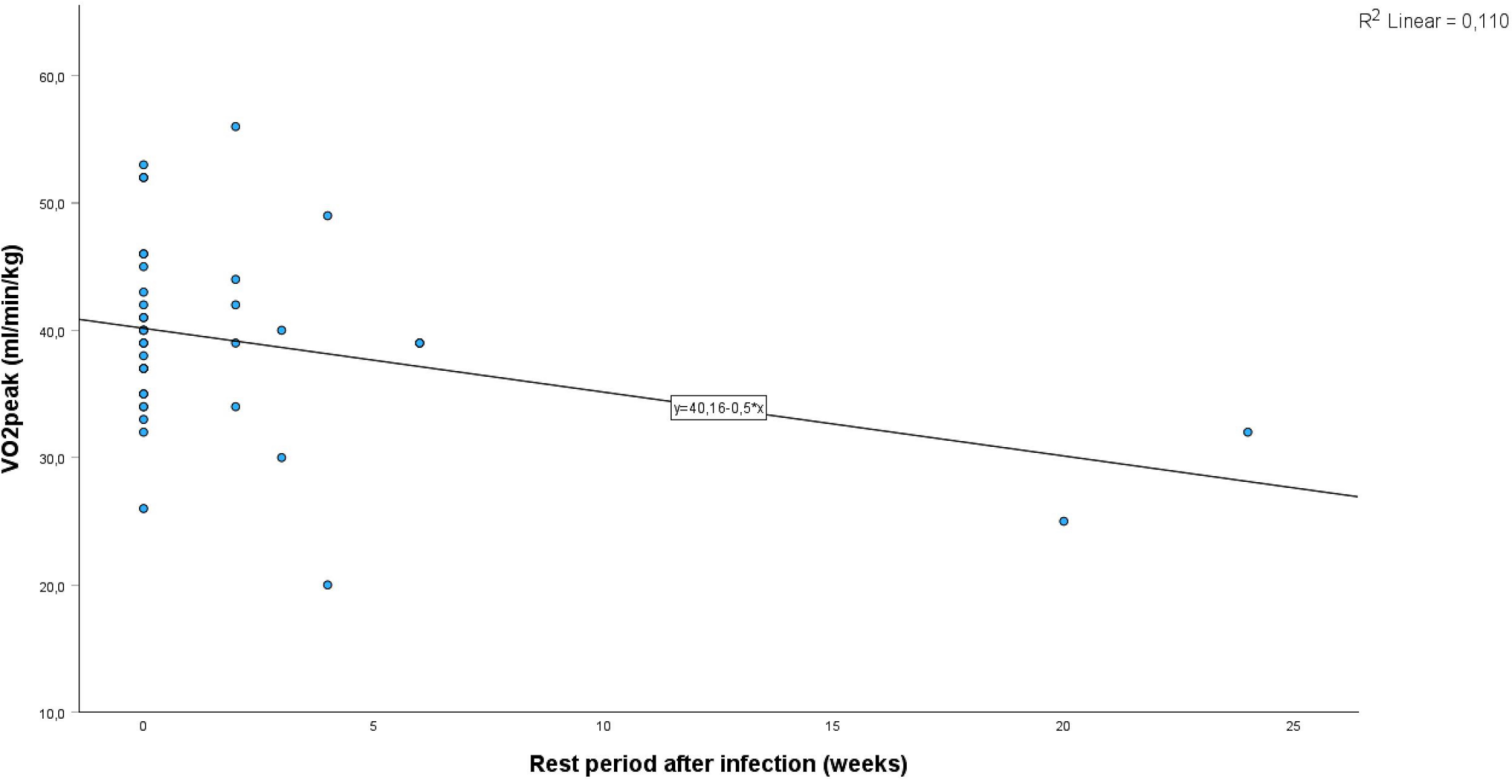
Correlation between the rest period after infection with SARS-CoV-2 in weeks and the $\dot{{\text{V}}}{{\text{O}}}_{2}{\text{peak}}$

When calculating possible correlations only one proved significant: the negative correlation between the duration of rest from physical activity after an infection with SARS-CoV-2 and the $\dot{{\text{V}}}{{\text{O}}}_{2}{\text{peak}}$ with a *p*-value of 0.044 (Fig. 2).

## Discussion

### Physical activity, exercise time and peak oxygen uptake

Interestingly, there were no significant differences between the two groups with regard to the amount of physical activity before the infection with SARS-CoV-2, nor in their school transport habits, or in their subjective exercise tolerance. The notion that premorbid $\dot{{\text{V}}}{{\text{O}}}_{2}{\text{peak}}$ may have been low in the post-COVID-19 group, one possible explanation for the previously lower values observed in the adult population, therefore seems unlikely [40]. However, after the infection, the participants in the post-COVID-19 group needed a longer rest period from physical activity than the control group (over 4 weeks vs. less than 1 week) and felt subjectively less exercise-tolerant. This is in accordance with previous survey studies investigating the effects of post-COVID-19 in children [6, 9–11, 13, 15].

Almost all participants achieved maximal exercise (defined as an RER > 1.00). There were also no differences with regard to heart rate recovery (HRR) or breathing reserve (BR). In previous studies in adults, the fact that HRR was lower in patients suffering from post-COVID-19 was taken as a sign of earlier termination of exercise due to deconditioning and fatigue [41]. Raman et al. [19] who also observed shorter walk distances in a 6-min-walk-test observed that many patients stopped CPET early because of generalized muscle ache rather than breathlessness. Interestingly, the majority of participants who suffered from post-COVID-19 in the current study also reported muscle fatigue as the main cause for stopping the exercise.

The absolute value of peak oxygen uptake was lower in the post-COVID19 group. This is in accordance with numerous studies conducted in the adult population [23, 24, 40] and reflects observations from survey and chart review studies conducted in the pediatric population [6, 9, 13–15]. However, when comparing the values for the percentage of predicted $\dot{{\text{V}}}{{\text{O}}}_{2}{\text{peak}}$ achieved, there was no significant difference. This could be a consequence of a relatively small sample size. Normal values for children represent an approximation and are gathered according to age. This leads to a smoothing of the results, which could therefore have annihilated the differences observed for $\dot{{\text{V}}}{{\text{O}}}_{2}{\text{peak}}$. Nor was there any significant difference in the oxygen uptake efficiency slope (OUES). These findings suggest that children affected by post-COVID-19 do not show a measurable impairment of their cardiopulmonary function, a fact that is underlined by values of 90% and more of their predicted values in both groups.

When differentiating the data according to sex, it became apparent that the lower $\dot{{\text{V}}}{{\text{O}}}_{2}{\text{peak}}$ as well as the lower percentage of predicted $\dot{{\text{V}}}{{\text{O}}}_{2}{\text{peak}}$ was limited to the girls. The fact that females seem to experience more pronounced symptoms for post-COVID-19 has been described before, using surveys and 6MWT [42, 43], although the pathomechanism behind this finding is yet unclear.

### Pulmonary function

The fact that there were no significant differences between the pulmonary variables (${\dot{{\text{V}}}}_{{\text{E}}}{\text{peak}}$, ${\dot{V}}_{{\text{E}}}$ at VT1, breathing reserve BR, breathing frequency BF) recorded during CPET between the two groups underlines the fact that pulmonary function does not seem to be the cause for exertional dyspnea or reduced exercise tolerance in children.

One possible explanation for dyspnea after COVID-19 is dysfunctional breathing [44]. A marker for ventilatory efficiency is the ventilatory equivalent (${\dot{V}}_{{\text{E}}}/{\dot{V}}_{{{\text{CO}}}_{2}}$-slope) [40]. Altered pulmonary diffusion capacity, ventilation/perfusion mismatch and hyperventilation-syndrome are possible causes for dysfunctional breathing and have been documented in COVID-19 survivors [45]. Interestingly the ventilatory equivalent between the two groups in our study did not differ significantly. Thus, ventilatory inefficiency seems an unlikely candidate for the subjective reduced exercise tolerance reported by children suffering from post-COVID-19.

### Cardiac function

Parameters for unmasking cardiovascular limitation using CPET are the O_2_pulse, the peak HR, or an abnormal increase of the $\dot{V}{{\text{O}}}_{2}/P$-slope. Despite the concerns around cardiac involvement during the SARS-CoV-2 pandemic, most studies showed normal values for the O_2_pulse in patients suffering from post-COVID-19 [23] or recovering from severe illness [46]. This was also true in this study.

At least a mild chronotropic incompetence has been observed in most studies conducting CPET in adults after infection with SARS-CoV-2 [46]. Lower peak HR was discussed either being due to chronotropic incompetence or a pharmaceutical betablockade or as a consequence of ceasing exercise early [41]. The children in this study did not show any significant differences with regard to chronotropic incompetence, and the HRR was well above the pathological 12 beats/minute [47]. This difference between the adult population and the children studied here may be due to several factors. First of all, the children showed a high willingness to reach peak exertion, reflected in the fact that almost all participants reached RER values above 1, irrespective of their symptoms, whereas the adults had probably ceased exercise before reaching peak exertional capacity, which may have been related to dyspnea unrelated to post-COVID-19 [41]. On the other hand, the use of beta-blocker was wide-spread in the investigated adult cohorts, also offering an explanation for the chronotropic incompetence. In contrast, none of the children involved in this study was on beta-blockers.

A further parameter which is typically reduced in patients with cardiovascular disease is the $\dot{{\text{V}}}{{\text{O}}}_{2}/P$-slope reflecting limitations in the supply and/or metabolism of oxygen. None of the children investigated in this study exhibited pathological values, defined as values below 10 ml/min/W [48]. This stands in contrast to studies investigating this parameter in the adult population, where the fact that it was slightly reduced was taken as a sign for a potential contribution of cardiovascular factors to the observed low $\dot{{\text{V}}}{{\text{O}}}_{2}{\text{peak}}$ [49].

### Peripheral function

The third compartment influencing $\dot{{\text{V}}}{{\text{O}}}_{2}{\text{peak}}$ is the periphery (musculature and mitochondria). Peripheral limitations can be assessed through an abnormal response in RER, abnormal $\dot{{\text{V}}}{{\text{CO}}}_{2}$-kinetics throughout exercise, a shallower $\dot{V}{O}_{2}/P$-slope or a reduced VT1 in relation to $\dot{{\text{V}}}{{\text{O}}}_{2}{\text{peak}}$. However, all of these parameters are unspecific and are also used to assess the cardiac compartment (see above) [40]. It is thus difficult to attribute abnormal findings in these parameters to deconditioning [40]. Some authors have therefore declared the observed reduction in $\dot{{\text{V}}}{{\text{O}}}_{2}{\text{peak}}$ to be a consequence of deconditioning in the absence of ventilatory and cardiac exercise limitations.

None of these parameters showed any significant difference between the group of children suffering from post-COVID-19 and those who did not. After completing the exercise, all children suffering from post-COVID-19 stated that they had to stop the exercise due to muscular fatigue, which was not the case in the comparison group. This observation suggests muscular deconditioning as the possible mechanism for the observed reduction in $\dot{{\text{V}}}{{\text{O}}}_{2}{\text{peak}}$.

Another parameter reintroduced by Longobardi et al. [24], the ${T}_{1/2}\dot{{\text{V}}}{{\text{O}}}_{2}$, is defined as the time needed for $\dot{{\text{V}}}{{\text{O}}}_{2}{\text{peak}}$ to decrease by half [24]. This value proved to be significantly longer in adults suffering from post-COVID-19, suggesting a slower replenishment of energy stores in peripheral muscles underlying this defective off-transient $\dot{{\text{V}}}{{\text{O}}}_{2}$ kinetic response [24]. Even though there were no significant differences between the two groups in this study, the children suffering from post-COVID-19 had values above a cut-off value of 90 s, which is generally assumed to be the upper limit of normal [24]. This supports the idea of a peripheral mechanism as the cause for the reduced exercise tolerance.

Interestingly, the only other significant difference between the two groups was the duration of rest after an infection with SARS-COV-2. The children suffering from post-COVID-19 stated a mean absence from physical activity of 4 weeks compared to 0.3 weeks in the comparison group. Furthermore, the only significant correlation of all the study parameters was between the duration of the rest period and $\dot{{\text{V}}}{{\text{O}}}_{2}{\text{peak}}$. A study investigating the effects of early deconditioning of human skeletal muscle found several deconditioning processes to be initiated within the first 5 days of hypoactivity [50]. A rest period of a mean of 4 weeks as observed in our cohort of post-COVID-19 children could explain the observed decrease of $\dot{{\text{V}}}{{\text{O}}}_{2}{\text{peak}}$ and abnormal ${T}_{1/2}\dot{{\text{V}}}{{\text{O}}}_{2}$. Since neither the pulmonary nor the cardiac compartment seems to be affected by infection with SARS-CoV-2, it seems reasonable to expect the cause in the peripheral compartment, i.e. the muscles. The combination of a significant difference between the two groups regarding the duration of rest with the negative correlation between rest period and $\dot{{\text{V}}}{{\text{O}}}_{2}{\text{peak}}$ suggests a connection between inactivity and the occurrence of post-COVID-19 symptoms. However, as this is a retrospective study, it is impossible to say whether the inactivity was the cause of the prolonged symptoms after SARS-CoV-2 infection or whether a more serious course of the infection leads to prolonged inactivity and prolonged symptoms at the same time. The fact remains that long rest periods before returning to sports as recommended with myocarditis [51] are probably not warranted as the heart seems unaffected. Instead we should encourage children after a Coronavirus infection to return to sports within the limits of an infectious disease[52] in order to improve the peripheral compartment and consequently the $\dot{{\text{V}}}{{\text{O}}}_{2}{\text{peak}}$. This recommendation obviously exclusively applies to children with post-COVID-19 syndrome and does not apply to children with paediatric inflammatory multisystem syndrome temporally associated with COVID-19 (PIMSts)/multisystem inflammatory syndrome in children (MIS-C) who need to be followed up as currently recommended [53].

## Conclusion

As observed previously in post-COVID-19 studies in the adult population, the absolute $\dot{{\text{V}}}{{\text{O}}}_{2}{\text{peak}}$ (but not the percentage of predicted $\dot{V}{O}_{2}peak$) was reduced in children suffering from post-COVID-19 symptoms. However, neither pulmonary, nor cardiac parameters proved to be significantly different from a group with no symptoms. The only other difference between the two groups was the rest period taken from physical activity after the infection with SARS-CoV-2. This suggests peripheral deconditioning as a possible cause for the reduction in cardiopulmonary function.

## Limitations

The study has several limitations. For one, the sample size was comparably small with 20 children in the post-COVID-19 group. Thankfully, the number of children suffering from post-COVID-19 is rather low, which made the recruitment challenging. However, many studies in the adult population are also limited to a comparable number of patients.

This study is a retrospective study. There is no data on the further course of post-COVID-19 in children.

## Data Availability

As a consequence of the small cohort, the data can only be made available for direct inquiries directed at the authors. Otherwise patient confidentiality cannot be ensured.

## Abbreviations

ANS: Autonomic nervous system
AWMF S1: Arbeitsgemeinschaft der Wissenschaftlichen Medizinischen Fachgesellschaften step 1
BPD: Bronchopulmonary dysplasia
BF: Breathing frequency
BR: Breathing reserve
CPET: Cardiopulmonary exercise test
HR: Heart rate
HRR: Heart rate recovery
OUES: Oxygen uptake efficiency slope
Peak HR: Peak heart rate
peakRER: RER at maximal exertion
RER: Respiratory exchange ratio
VCO_2_: Carbon dioxide elimination
VE: Minute ventilation
VEpeak: Peak minute ventilation
*V*_E_/*V*_CO2_: Breathing efficiency
VO_2_: Oxygen uptake
VO_2_ peak: Peak oxygen uptake
vpeak: Peak speed
VT1: Ventilatory threshold 1
VT2: Ventilatory threshold 2
